# Differential roles of VPS and RAAS in water homeostasis and a risk for kidney dysfunction in rats undergoing rapid fasting/dehydration with regular exercise

**DOI:** 10.14814/phy2.14670

**Published:** 2021-01-05

**Authors:** KazuyA Hasegawa, Yuya Yamaguchi, Masashi Tanaka

**Affiliations:** ^1^ Faculty of Nutritional Sciences Morioka University Takizawa city Japan; ^2^ Department of Physiology Faculty of Medicine Toho University Japan; ^3^ Department of Physical Therapy Health Science University Minamitsuru‐gun Japan

**Keywords:** acute dehydration, body fluid homeostasis, exercise, renal inflammation, sodium reabsorption

## Abstract

**Purpose:**

We examined the effects of rapid restriction of food and fluid intake on the pathways of water homeostasis, the vasopressinergic system (VPS), and the renin–angiotensin–aldosterone system (RAAS), in rats with or without regular exercise.

**Methods:**

Sprague Dawley rats were divided into the following groups: no intervention, rapid restriction, regular exercise, and rapid restriction combined with regular exercise. Rats in the exercise group performed climbing exercise for 4 weeks. All rats consumed food ad libitum, and those in the rapid restriction group fasted for the last 3 days with no water on the last 1 day.

**Results:**

Despite no significant differences in body weight among the groups, the kidney weight was decreased when rapid restriction and regular exercise were combined. Rapid restriction reduced the urine volume and increased the urine osmolality, whereas regular exercise did not. Rapid restriction but not regular exercise increased the levels of circulating aldosterone and the renal expression levels of the ion channel SGK‐1 compared to those without rapid restriction, indicating the stimulation of RAAS. Conversely, VPS showed no significant response to these interventions. Moreover, rapid restriction combined with regular exercise induced the renal expression levels of proinflammatory cytokines and increased the active forms of apoptotic effector caspase‐3 compared with the no intervention group.

**Conclusions:**

Functional significance may differ between VPS and RAAS in water homeostasis in response to rapid restriction. Moreover, the combination of rapid restriction and regular exercise has potentially deleterious effects on the kidney.

## Graphical abstract

The mechanistic details underlying water homeostasis in response to rapid dehydration, with and without regular exercise, have not been elucidated. Our data revealed that Renin–angiotensin–aldosterone system was stimulated by rapid restriction, whereas vasopressin was not, suggesting the novel differential functional significance of these pathways in water homeostasis.

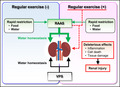

## 1 INTRODUCTION

Rapid weight loss in athletes is one of the methods to reduce body weight (BW), which is characterized by a transient weight loss of at least 5% of BW within a week (Khodaee et al., 2015). This is performed by several methods such as the reduction in food and fluid intake and the increase in body secretions (Franchini et al., 2012; Khodaee et al., 2015; Reale et al., 2017; Silva Santos et al., 2016). It has been hypothesized that, in weight‐sensitive sports, rapid weight loss could confer a higher competitive edge to the subjects compared with those originally belonging to the weight‐class of interest without weight reduction, if rapid weight loss could maximize the loss of fat and water but minimize the loss of glycogen and muscle (Fernández‐Elías et al., 2014; Khodaee et al., 2015; Pettersson & Berg, 2014; Reljic et al., 2013). In humans, in particular, approximately 65% of the body consists of water, and it might be a potential target of rapid weight loss (Khodaee et al., 2015); however, rapid dehydration exceeding 5% of BW elevates the risk for adverse health outcomes, including muscle injury, acute kidney injury, heart attack, and death (Centers for Disease Control and Prevention (CDC), 1998; Franchini et al., 2012; Khodaee et al., 2015; Pettersson & Berg, 2014; Kasper et al., 2018). Despite these pathological relationships between rapid dehydration and clinical outcomes, the mechanistic details underlying water homeostasis in response to rapid dehydration, with and without regular exercise, remain to be elucidated.

The maintenance of water homeostasis involves various factors, including endocrine, nervous, and hemodynamic systems (Bai et al., 2017). In endocrine systems, two canonical pathways play major roles in water homeostasis by regulating the renal function. One is the vasopressinergic system (VPS) in which the antidiuretic hormone arginine vasopressin (AVP) is secreted from the posterior pituitary gland into the circulation in response to physiological changes such as serum osmolality elevation and hypovolemia. AVP binds to its receptors and stimulates the expression and trafficking of water channel aquaporin 2 (AQP2), thereby leading to increased water reabsorption and concomitant water retention in the kidney (Bai et al., 2017; Qian, 2018; Szczepanska‐Sadowska et al., 2018). The second pathway is the renin–angiotensin–aldosterone system (RAAS) that stimulates the release of aldosterone, the mineralocorticoid hormone secreted by the adrenal gland in response to changes in fluid volume and/or sodium concentration in the blood. Aldosterone increases the expression and stability of the epithelial sodium channel (ENaC), which is responsible for sodium retention, thereby contributing to water retention in the kidney (Bai et al., 2017; Szczepanska‐Sadowska et al., 2018). Both VPS and RAAS are influenced by a major renal metabolite, prostaglandin E2 (PGE_2_), which is produced by a sequential reaction of enzymes, including cyclooxygenase 2 (COX‐2) and microsomal PGE synthase‐1 (mPGES‐1) (Li et al., 2017). Reportedly, PGE_2_ negatively regulates the reabsorption of water by VPS and of sodium by RAAS (Li et al., 2017; Nasrallah et al., 2018). In addition, recent accumulating evidence suggests that the cross‐talk between VPS and RAAS also exerts regulatory effects on the water–electrolyte balance (Szczepanska‐Sadowska et al., 2018).

Regarding the potential effects of exercise on water homeostasis, it has been reported that exercise training reduced the serum levels of AVP and the renal expression levels of AQP2 in a rat model of chronic heart failure (Lin et al., 2011). Another study reported that acute exercise resulted in an increase in plasma aldosterone levels through RAAS activation in normal rats (Lieu et al., 2014). Conversely, in another rat model study of unilateral renal artery stenosis, daily exercise reduced plasma aldosterone levels, thereby leading to a lower arterial pressure without detrimental effects on renal function (Waldman et al., 2017). These findings suggest that exercise has some effects on the activities of VPS and/or RAAS; however, the functional significance of VPS and RAAS in water homeostasis under rapid dehydration with and without regular exercise has not yet been clarified.

Therefore, in the present study, we investigated the effects of rapid restriction of food and fluid intake on the activities of VPS and RAAS in rats with and without regular exercise. Our findings demonstrated the differential functional significance of VPS and RAAS in water homeostasis under the rapid restriction of consumption.

## 2 MATERIALS AND METHODS

### 2.1 Animals

Male Sprague Dawley rats aged 5 weeks were purchased from Japan SLC (Hamamatsu, Japan). The rats were maintained in a facility with free access to water and standard chow (CE‐2, CLEA Japan, Tokyo, Japan) under a 12:12 hr light–dark cycle at 22°C–24°C. To examine the effects of exercise and rapid restriction of food and water intake, the rats were randomly divided into the following four groups: nonexercise without rapid restriction, nonexercise with rapid restriction, exercise without rapid restriction, and exercise with rapid restriction (*n* = 5 in each group). All animal experiments were approved by the Animal Research Committee and were conducted in accordance with the Morioka University Guidelines for Animal Experiments.

### 2.2 Study design

As a regular exercise program, rats in the exercise group were subjected to a climbing exercise, which has been previously used to investigate the effects of the rapid restriction of food and water on the body composition of rats (Tai et al., 2009, 2010). Briefly, the climbing exercise apparatus had a wire mesh cage (length 40 × width 30 × height 60 cm) placed on a 53°C electric hot plate. This was the minimum temperature required to stimulate the rats to climb the cage whenever they touched the bottom plate during the exercise session, and the hot plate was used to allow rats in the exercise group to perform the exercise. After 5 days to make the rats familiar with the wire cage, they were subjected to an exercise program consisting of two sets of a 15‐min exercise per day, which also included a 15‐min rest between the two exercise sessions. This exercise program was conducted for 4 weeks (5 days/week) with a focus on the protection of rats from external injuries. Rats in the rapid restriction group (with and without the above‐described exercise) were fasted for 3 days, with no provision of water on the last 1 day. All rats were euthanized under isoflurane anesthesia, and plasma, hypothalamus tissue, and kidney medulla were collected and stored at −80°C.

### 2.3 Urine samples

The rat cage had a wire mesh floor on hydrophobic sand (LabSand, Braintree Scientific, MA, USA), which was spread on the bottom to collect urine samples (Hoffman et al., 2017, 2018). Urine on the surface of the sand was collected using a pipette at 6‐hr intervals till the last 12 hr before sacrifice. The total volume of urine samples was measured, and urine osmolality was calculated from the freezing point depression at Morioka Clinical Laboratory Center, Inc. (Iwate, Japan).

### 2.4 Quantification of biomarkers in the blood

Biomarkers of interest in the blood were quantified by a colorimetric assay using a relevant commercially available kit, according to the manufacturer's instructions, as follows: plasma sodium (Na^+^) concentration, a Sodium assay kit (SIGMA‐ALDRICH, St. Louis, MO, USA); blood urea nitrogen (BUN), a Urea nitrogen (BUN) colorimetric detection kit (Arbor Assays, Ann Arbor, MI, USA); plasma creatinine concentration, a Creatinine (serum) colorimetric assay kit (Cayman Chemical, Ann Arbor, MI, USA); level of vasopressin in plasma, an Arg^8^‐Vasopressin ELISA kit (Enzo Life Sciences, Farmingdale, NY, USA); and level of aldosterone in plasma, an Aldosterone ELISA kit (Enzo Life Sciences).

### 2.5 Total RNA extraction and quantitative RT‐PCR

Total RNA was separately extracted from the hypothalamus or kidney medulla using Sepasol Reagent (Nacalai Tesque, Kyoto, Japan), and first‐strand cDNA was synthesized using a PrimeScript RT Master Mix (Takara Bio, Shiga, Japan), according to the manufacturer's instructions. The expression levels of the genes of interest were measured by quantitative RT‐PCR using SYBR Premix Ex Taq II reagents (Takara Bio) and the Step One Real‐time PCR system (Applied Biosystems, Foster City, CA, USA). The expression levels of glyceraldehyde‐3‐phosphate dehydrogenase (GAPDH) were used as internal control. The following primer sequences were used: rat heteronuclear AVP forward primer (5′‐GAGGCAAGAGGGCCACATC‐3′), reverse primer (5′‐CTCTCCTAGCCCATGACCCTT‐3′) (Greenwood et al., 2015); rat mature AVP forward primer (5′‐TGCCTGCTACTTCCAGAACTGC‐3′), reverse primer (5′‐AGGGGAGACACTGTCTCAGCTC‐3′) (Greenwood et al., 2015); rat serum‐ and glucocorticoid‐inducible kinase‐1 (SGK‐1) forward primer (5′‐TAGCAATCCTCATCGCTTTC‐3′), reverse primer (5′‐GAGTTGTTGGCAAGCTTCTG‐3′) (Wong et al., 2007); rat AQP2 forward primer (5′‐GGACCTGGCTGTCAATGCTC‐3′), reverse primer (5′‐GCGGGCTGGATTCATGGAG‐3′) (Wang et al., 2015); rat AQP3 forward primer (5′‐CCCCTTGTGATGCCTCTC‐3′), reverse primer (5′‐CCCTAGCTGGCAGAGTTC‐3′); rat AQP4 forward primer (5′‐AGGAGGACCCAGGCAATG‐3′), reverse primer (5′‐GGCAAGGTCTCATGCCATC‐3′); rat glucocorticoid‐induced leucine zipper (GILZ) forward primer (5′‐CGGCAACCCGAATCATGAAC‐3′), reverse primer (5′‐TGGCTCCAGAGGCACTGTTA‐3′); rat corticosteroid hormone‐induced factor (CHIF) forward primer (5′‐GGGAATAACCTGTGCCTTTC‐3′), reverse primer (5′‐AGGGACTGCCTTTATCAACTG‐3′) (Wong et al., 2007); rat ENaCα forward primer (5′‐ACATTCTGTCCAGGCTGTCG‐3′), reverse primer (5′‐TGGAATAATTCGCCTGGTTGC‐3′); rat COX‐2 forward primer (5′‐ACCAACGCTGCCACAACT‐3′), reverse primer (5′‐GGTTGGAACAGCAAGGATTT‐3′) (Hasegawa et al., 2015); rat mPGES‐1 forward primer (5′‐GGCTGGCTAGCTGAGATGAC‐3′), reverse primer (5′‐TCCACATCTGGGTCACTCCT‐3′); rat tumor necrosis factor‐alpha (TNF‐α) forward primer (5′‐ATCGGTCCCAACAAGGAGGA‐3′), reverse primer (5′‐CTCCGCTTGGTGGTTTGCTA‐3′) (Hasegawa et al., 2015); rat interleukin (IL)‐1β forward primer (5′‐CCTTGTGCAAGTGTCTGAAG‐3′), reverse primer (5′‐GGGCTTGGAAGCAATCCTTA‐3′); rat IL‐6 forward primer (5′‐AGAAAAGAGTTGTGCAATGGCA‐3′), reverse primer (5′‐GGCAAATTTCCTGGTTATATCC‐3′); rat kidney injury molecule‐1 (Kim‐1) forward primer (5′‐CCACAAGGCCCACAACTATT‐3′), reverse primer (5′‐TGTCACAGTGCCATTCCAGT‐3′) (Nielsen et al., 2017); rat neutrophil gelatinase‐associated lipocalin (Ngal) forward primer (5′‐GATCAGAACATTCGTTCCAA‐3′), reverse primer (5′‐TTGCACATCGTAGCTCTGTA‐3′) (Nielsen et al., 2017); and rat GAPDH forward primer (5′‐TGACCTCAACTACATGGTCTACA‐3′), reverse primer (5′‐CTTCCCATTCTCGGCCTTG‐3′) (Wang et al., 2015) (Greiner Bio‐One, Tokyo, Japan).

### 2.6 Western blot analyses

Kidney medulla from the rats was resuspended in RIPA lysis buffer (Wako, Osaka, Japan) supplemented with protease and phosphatase inhibitor cocktails (Nacalai Tesque). The total proteins (AQP2, AQP3, and AQP4 detection, 5 µg; caspase‐3, 20 µg) were resolved by SDS‐polyacrylamide gel electrophoresis (AQP2, 10%; AQP3 and AQP4, 5%–20%; caspase‐3, 15%) and transferred to polyvinylidene difluoride membranes, respectively. After blocking with a blocking solution (AQPs, 5% skimmed milk; caspase‐3, Blocking One [Nacalai Tesque]), the membranes were incubated overnight with the rabbit primary antibody anti‐AQP2 (1:5,000 dilution) (Alomone Labs, Jerusalem, Israel), anti‐AQP3 (1:10,000 dilution) (Alomone Labs), anti‐AQP4 (1:10,000 dilution) (Alomone Labs), or anti‐caspase‐3 (1:2000) (Cell Signaling Technology, Beverly, MA, USA) at 4°C with gentle shaking. The immunoreactive bands were detected with an HRP‐conjugated goat anti‐rabbit IgG secondary antibody (Southern Biotech, Birmingham, AL, USA) and the ECL Prime Western Blotting Detection Reagent (GE Healthcare, Little Chalfont, England). For internal control, the membranes were blocked with 5% skimmed milk, followed by incubation with anti‐β‐actin antibody (1:2000) (Proteintech, IL, USA), and then the immunoreactive band was detected as described above. Gel images were acquired using the ImageQuant LAS 4000 (GE Healthcare), and protein levels were quantified by measuring the band intensities using ImageJ (NIH, Bethesda, MD, USA). Regarding AQP2 and AQP3 proteins, two signals corresponding to the nonglycosylated (the lower molecular weight) and glycosylated (the higher molecular weight) forms were detected so that each signal was quantified and the sum of the two values was compared between the groups, as reported previously (Baggaley et al., 2010).

### 2.7 Statistical analysis

Data are expressed as mean ± *SEM*. Mean values were compared between the groups by one‐way ANOVA with Tukey's post hoc tests (Tukey–Kramer test) for pairwise comparisons using GraphPad Prism 6.0 (GraphPad Software Inc., San Diego, CA, USA). Two‐tailed *p* < .05 was considered to be statistically significant.

## 3 RESULTS

### 3.1 Effects of the rapid restriction of food and water intake on BW, KW, body fluid tonicity, and blood biomarkers with and without regular exercise

**Figure 1:**
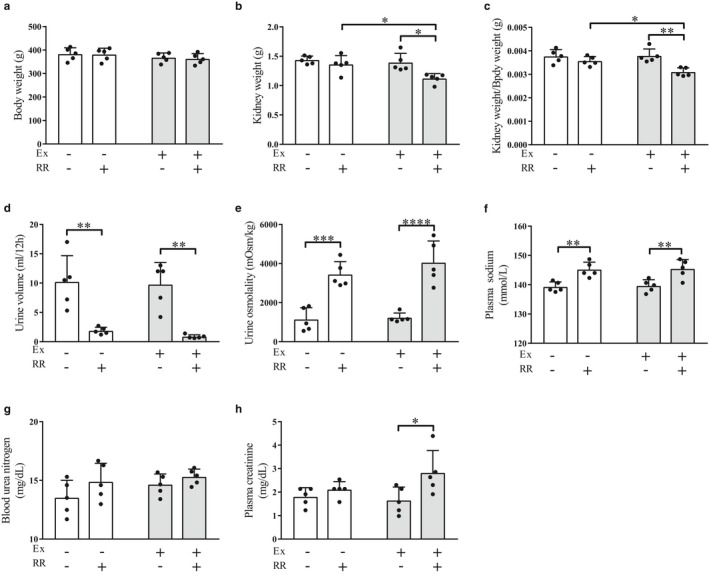
Effects of the rapid restriction of food and water intake on body weight, kidney weight, urine volume, urine osmolality, and blood biomarkers of rats with and without regular exercise. (a) Body weight of rats at the end of the study period. (b) Weight of kidney obtained from rats at the end of the study period. (c) The ratio of kidney weight to body weight. (d) Volume of urine samples collected during the last 12 hr before sacrifice. (e) Osmolality of urine samples obtained in (d). The osmolality was calculated from the freezing point depression. (f) Plasma Na^+^ concentration. (g, h) Concentrations of blood urea nitrogen (g) and plasma creatinine (h). Data are expressed as mean ± *SEM* (*n* = 5/group). *p < .05; **p < .01; ***p < .001; ****p < .0001. NS, not significant; Ex, exercise; RR, rapid restriction

To elucidate the mechanisms underlying water homeostasis, we first examined the effects of the rapid restriction of food and water intake on body fluid tonicity of rats with and without regular exercise. We found that, in the nonexercise group, rapid restriction had no substantial effects on BW and kidney weight (KW) as well as on the ratio of KW to BW under our experimental conditions (Figure 1a–c). In contrast, the urine volume in the nonexercise group was significantly decreased by the rapid restriction (Figure 1d, *p* < .01) in conjunction with the significant increase in urine osmolality (Figure 1e, *p* < .001). Rapid restriction also significantly elevated plasma Na^+^ concentration (Figure 1f, *p* < .01), whereas it had no significant influence on the concentrations of BUN and plasma creatinine, which are blood markers of renal function (Figure 1g,h).

In the exercise group, rapid restriction had no significant effect on BW, as observed in the nonexercise group (Figure 1a). However, rapid restriction significantly reduced the KW as well as the ratio of KW to BW in this group (Figure 1b, *p* < .05; Figure 1c, *p* < .01). Moreover, both KW and KW/BW ratio were significantly lower in the exercise group than in the nonexercise group (Figure 1b,c, *p* < .05); these results suggest that rapid restriction has some potential pathological effects on the renal function of rats in the group that received regular exercise. Rapid restriction in the exercise group also exhibited significant effects on the urine volume and osmolality (Figure 1d and e, *p* < .01 and *p* < .0001, respectively) and on plasma Na^+^ concentration (Figure 1f, *p* < .01), as observed in the nonexercise group (Figure 1d–f). The concentration of BUN was not significantly affected (Figure 1g), but that of plasma creatinine was significantly elevated (Figure 1h, *p* < .05), by rapid restriction combined with regular exercise.

### 3.2 Effects of the rapid restriction of food and water intake on the levels of AVP with and without regular exercise

**Figure 2:**
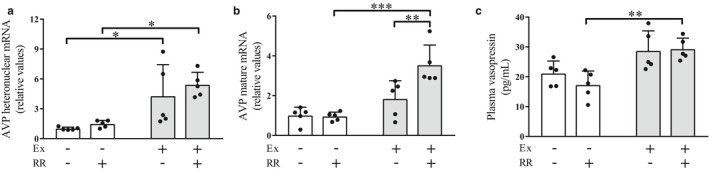
Effects of the rapid restriction of food and water intake on vasopressin levels in rats with and without regular exercise. (a) Expression levels of AVP heteronuclear RNA in the hypothalamus. RNA expression levels were analyzed by quantitative RT‐PCR and normalized to those of GAPDH. Fold changes are displayed relative to the control group receiving neither regular exercise nor rapid restriction (1.0). (b) Expression levels of AVP mature mRNA in the hypothalamus. Data are normalized and displayed as described in (a). (c) Circulating levels of vasopressin. The amounts of vasopressin in the plasma were quantified by ELISA. Data are expressed as mean ± *SEM* (*n* = 5/group). *p < .05; **p < .01. Ex, exercise; RR, rapid restriction

We next investigated the effects of the rapid restriction of food and water intake on the AVP–AQP axis, the pathway for regulating water balance, in the nonexercise and exercise groups. In the nonexercise group, we observed that rapid restriction had no significant effect on the hypothalamic expression levels of AVP as well as on the circulating levels of AVP (Figure 2a–c). Similarly, it had no significant effect on the hypothalamic expression levels of heteronuclear AVP (Figure 2a) and on the circulating levels of AVP (Figure 2c) in the exercise group, except that the hypothalamic expression levels of mature AVP were significantly elevated by rapid restriction (Figure 2b, *p* < .01). These results suggest that rapid restriction itself had a minor role in regulating the AVP levels in the nonexercise and exercise groups. Conversely, when rapid restriction was combined with regular exercise, both the hypothalamic expression levels and the circulating levels of AVP were significantly elevated compared with those in the rapid restriction without regular exercise group (Figure 2a, *p* < .05; Figure 2b, *p* < .001; Figure 2c, *p* < .01). Therefore, these findings suggest that the combination of rapid restriction and regular exercise positively regulates the production of AVP.

### 3.3 Effects of the rapid restriction of food and water intake on the levels of renal water channels under the conditions with and without regular exercise

**Figure 3:**
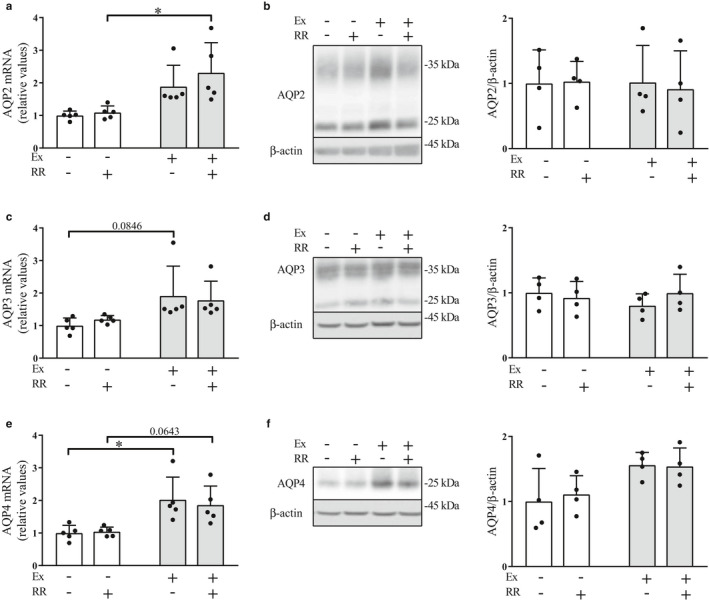
Effects of the rapid restriction of food and water intake on the renal expression levels of water channels in rats with and without regular exercise. (a) Gene expression levels of AQP2 in the kidney medulla. The mRNA levels of AQP2 were obtained by quantitative RT‐PCR and normalized to those of GAPDH. Expression levels are displayed relative to those of the control group receiving neither regular exercise nor rapid restriction (1.0). (b) Protein levels of AQP2 in the kidney medulla. The amounts of the nonglycosylated (29 kDa) and glycosylated form (35 kDa) forms of AQP2 protein were analyzed by Western blot (left panel) and densitometry, and the sum of the two values was compared between the groups (right panel). The image shows representative immunoblots. (c–f) Gene expression levels and protein levels of AQP3 (c and d, respectively) and AQP4 (e and f, respectively) in the kidney medulla. Data are obtained and displayed as described in (a and b). Data are expressed as mean ± *SEM* (mRNA levels, *n* = 5/group; protein levels, *n* = 4/group). *p < .05. NS, not significant; Ex, exercise; RR, rapid restriction

We evaluated the renal expression levels of the effectors involved in the regulation of water homeostasis, that is., water channels AQP2, AQP3, and AQP4, among which AQP2 is particularly regulated by AVP (Kortenoeven & Fenton, 2014). Rapid restriction had no significant effect on the renal expression levels of these channels in either the nonexercise or the exercise group (Figure 3a–f). Conversely, rapid restriction significantly elevated the gene expression levels of AQP2 in the exercise group compared to those in the nonexercise group (Figure 3a, *p* < .05), whereas there was no significant difference in the protein levels of AQP2 between these groups (Figure 3b). Moreover, the expression levels of AQP3 showed no significant difference between the nonexercise and exercise groups (Figure 3c,d). In addition, rats in the exercise group without rapid restriction and with rapid restriction showed significant and near‐significant elevations in the expression levels of AQP4, respectively, compared with those in the nonexercise group (Figure 3e, *p* < .05 and *p* = .0643), but the protein levels of AQP4 did not differ significantly between the groups (Figure 3f). These results suggest a minor role of rapid restriction and regular exercise in the regulation of renal expression of these water channels, although AVP levels were affected by rapid restriction and/or regular exercise (Figure 2).

### 3.4 Effects of the rapid restriction of food and water intake on the levels of aldosterone under the conditions with and without regular exercise

**Figure 4:**
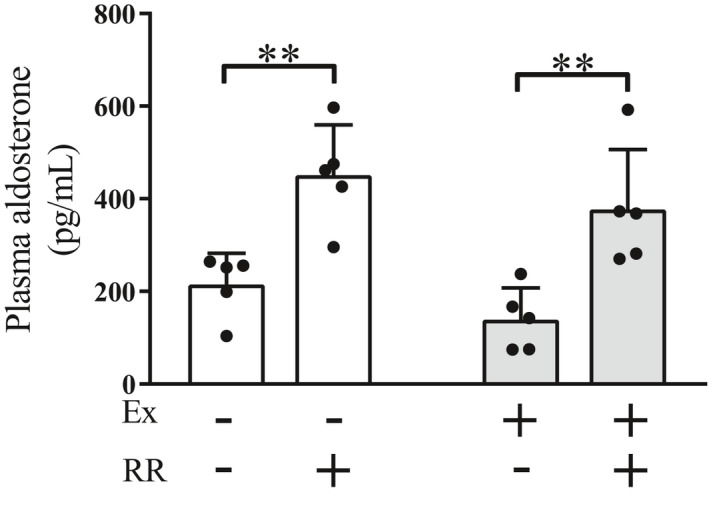
Effects of the rapid restriction of food and water intake on the circulating levels of aldosterone in rats with and without regular exercise. The amounts of aldosterone in the plasma were measured by ELISA. Data are expressed as mean ± *SEM* (*n* = 5/group). **p < .01. Ex, exercise; RR, rapid restriction

RAAS is another pathway for water homeostasis through the regulation of electrolyte balance. To examine the effects of rapid restriction with and without regular exercise on this pathway, we investigated the plasma levels of aldosterone, which acts as an effector molecule in renal electrolyte regulation. In contrast to the effects of rapid restriction on AVP levels (Figure 2), the plasma aldosterone levels were significantly elevated by rapid restriction in both the nonexercise and exercise groups (Figure 4, *p* < .01), consistent with the elevation of plasma Na^+^ concentration (Figure 1f). These findings suggest that rapid restriction exerts stimulatory effects on RAAS, irrespective of the absence or presence of regular exercise, which differs from the effects of rapid restriction on the levels of AVP.

### 3.5 Effects of the rapid restriction of food and water intake on the expression levels of ion channels with and without regular exercise

**Figure 5:**
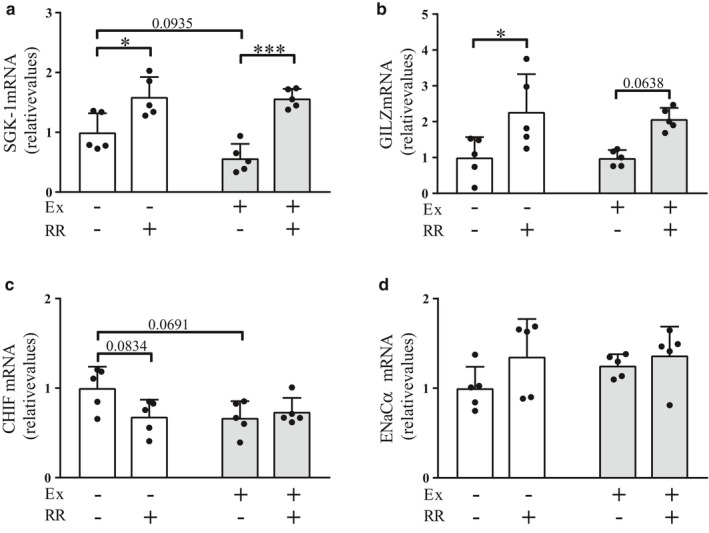
Effects of the rapid restriction of food and water intake on the renal expression levels of ion channels in rats with and without regular exercise. (a–d) Gene expression levels of SGK‐1 (a), GILZ (b), CHIF (c), and ENaCα (d) in the kidney medulla. The mRNA levels were analyzed by quantitative RT‐PCR and normalized to those of GAPDH. Fold changes are displayed relative to the control group receiving neither regular exercise nor rapid restriction (1.0). Data are expressed as mean ± *SEM* (*n* = 5/group). *p < .05; ***p < .001. NS, not significant; Ex, exercise; RR, rapid restriction

Next, we investigated the effects of rapid restriction on the renal expression levels of the ion channels SGK‐1 and GILZ in the nonexercise and exercise groups, as the expression levels of these channels have been reported to be elevated by aldosterone to stimulate the ENaC‐mediated sodium transport (Bhalla et al., 2006). Rapid restriction significantly upregulated the expression levels of SGK‐1 in both the nonexercise and exercise groups (Figure 5a, *p* < .05 and *p* < .001, respectively). It also resulted in significant and near‐significant elevations in the expression levels of GILZ in the nonexercise (*p* < .05) and exercise (*p* = .0638) groups, respectively (Figure 5b), thereby suggesting the elevation of sodium reabsorption. No significant effects of rapid restriction were found on the expression levels of CHIF, a regulator of Na^+^, K^+^‐ATPase (Geering, 2006), as well as of ENaCα per se, in both the nonexercise and exercise groups (Figure 5c,d).

### 3.6 Effects of the rapid restriction of food and water intake on the expression levels of enzymes for PGE_2_ production with and without regular exercise

We next examined whether rapid restriction and/or regular exercise affected the gene expression levels of COX‐2 and mPGES‐1, enzymes for PGE_2_ production; this metabolite is involved in suppressing water and sodium reabsorption (Li et al., 2017; Nasrallah et al., 2018). Results showed that the expression levels of COX‐2 were significantly elevated by rapid restriction in both the nonexercise and exercise groups (see Figure, Supplemental Digital Content [SDC] 1a, *p* < .01 and *p* < .0001, respectively). Moreover, the combination of rapid restriction and regular exercise resulted in the highest expression levels of COX‐2 between the groups (SDC 1a). Similar gene expression profiles were obtained for mPGES‐1 (SDC 1b). Therefore, these findings suggest that rapid restriction combined with regular exercise particularly enhanced the production of PGE_2_.

### 3.7 Effects of the rapid restriction of food and water intake on the levels of proinflammatory cytokines, apoptotic cell death, and tissue injury in the kidney with and without regular exercise

**Figure 6:**
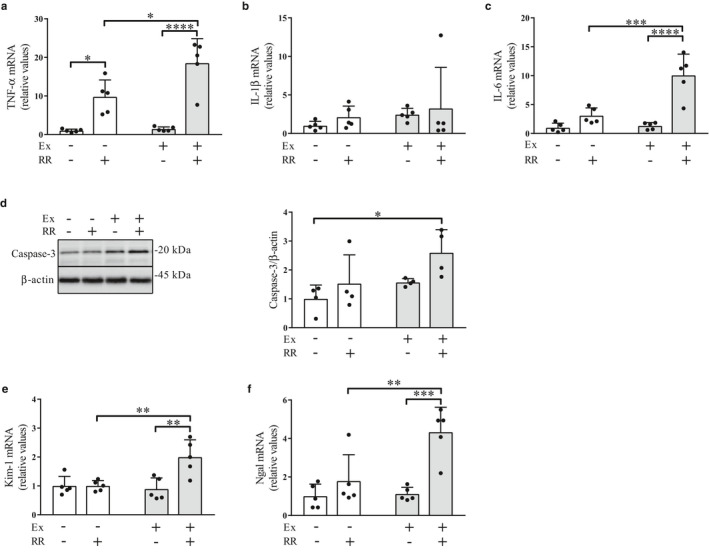
Effects of rapid restriction of food and water intake on the levels of inflammation, activation of an apoptotic effector, and levels of tissue injury in the kidney of rats with and without regular exercise. (a–c) Gene expression levels of TNF‐α (a), IL‐1β (b), and IL‐6 (c) in the kidney medulla. The mRNA levels were obtained using quantitative RT‐PCR and normalized to those of GAPDH. Fold changes are displayed relative to the control group receiving neither regular exercise nor rapid restriction (1.0). (d) Protein levels of active caspase‐3 in the kidney medulla. The amounts of active caspase‐3 protein were analyzed via Western blot (left panel) and densitometry (right panel). The image shows representative immunoblots. (e and f) Gene expression levels of Kim‐1 (e) and Ngal (f) in the kidney medulla. Data are expressed as mean ± *SEM* (mRNA levels, *n* = 5/group; protein levels, *n* = 4/group). *p < .05; **p < .01; ***p < .001; ****p < .0001. NS, not significant; Ex, exercise; RR, rapid restriction

As described earlier, rapid restriction with regular exercise reduced KW and the KW/BW ratio (Figure 1). Therefore, we investigated whether rapid restriction was implicated in renal cytotoxicity. We observed that in both the nonexercise and exercise groups, rapid restriction resulted in a significant elevation of the gene expression levels of the proinflammatory cytokine TNF‐α (Figure 6a, *p* < .05 and *p* < .0001, respectively). In addition, the combination of rapid restriction and regular exercise led to significantly higher expression levels of TNF‐α than those in the group subjected to rapid restriction alone (Figure 6a, *p* < .05) and the group that had neither rapid restriction nor regular exercise (Figure 6a, *p* < .0001). The expression levels of IL‐1β were not significantly different between the groups (Figure 6b), but those of IL‐6 in the exercise group were elevated by rapid restriction compared to those in the group without rapid restriction (Figure 6c, *p* < .0001). The levels of IL‐6 in the rapid restriction group under regular exercise were also higher than those in the nonexercise group without rapid restriction (Figure 6c, *p* < .0001). We further found that rats in the exercise group with rapid restriction showed a significant increase in the levels of the formation of the active apoptotic effector caspase‐3 compared to those in the no intervention group (Figure 6d, *p* < .05). In addition, rapid restriction with regular exercise significantly elevated the expression levels of Kim‐1 and Ngal, which are markers for the early detection of kidney injury (Devarajan, 2011, 2020; Uni et al., 2020) (Figure 6e, *p* < .01; Figure 6f, *p* < .01 and *p* < .001). These results suggest that rapid restriction under regular exercise exhibits deleterious effects on the renal cells, potentially by stimulating an inflammatory response.

## 4 DISCUSSION

**Figure 7:**
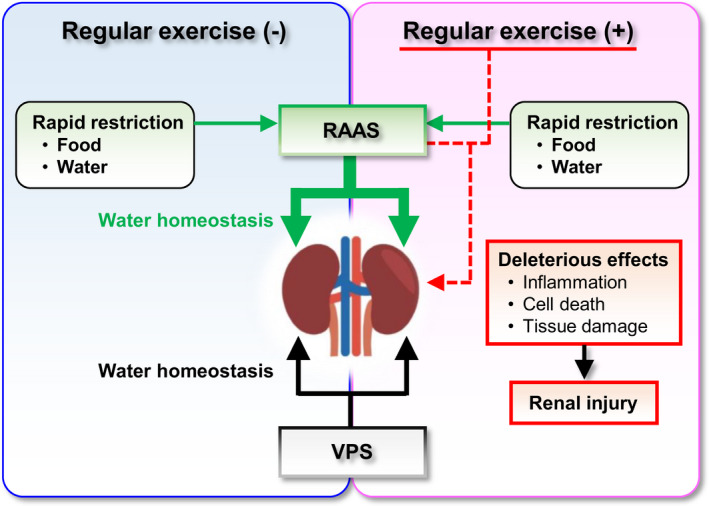
Effects of the rapid restriction of food and water intake on the pathways for water homeostasis in rats with and without regular exercise. The VPS regulates water homeostasis to the extent of the steady‐state conditions, irrespective of the presence or absence of regular exercise/rapid restriction. Conversely, the RAAS responds to rapid restriction and contributes to water homeostasis to deal with body fluid‐related acute stress. When rapid restriction is loaded in combination with regular exercise, some factors derived from the combination of these two stresses would exhibit deleterious effects on kidney function. VPS, vasopressinergic system; RAAS, renin–angiotensin–aldosterone system

This study has provided the first evidence demonstrating that RAAS but not VPS responded to the rapid restriction of food and fluid intake in rats, irrespective of whether they underwent regular exercise or not. In particular, the combination of rapid restriction and regular exercise elevated the levels of renal inflammation, formation of active apoptotic effectors, and levels of early markers for kidney injury, in parallel with the reductions in KW and KW/BW ratio and the increase in plasma creatinine concentration. These findings suggest the differential functional significance of VPS and RAAS in water homeostasis and further imply the potentially deleterious effects of rapid restriction combined with regular exercise on kidney function (Figure 7).

In the present study, we found that the expression levels of AVP and the circulating levels of vasopressin were affected by regular exercise and/or rapid restriction, which is consistent with previous reports that AVP levels were elevated by stresses, including exercise, which causes the increase in plasma osmolality (Coiro et al., 2011; Takamata et al., 2000). However, regarding the protein levels of AQP2 in the kidney, there was no significant effect of regular exercise and/or rapid restriction. If VPS would modulate water homeostasis to deal with regular exercise and/or rapid restriction, the protein levels of AQP2 in the kidney as well as the circulating levels of vasopressin would be elevated by these factors. Therefore, our results suggest that VPS through the AVP–AQP axis would constitutively regulate water homeostasis at steady‐state levels, even under the conditions of regular exercise and/or rapid restriction. Conversely, we found that RAAS was stimulated by rapid restriction, whereas regular exercise per se had no significant effect on RAAS. Therefore, these findings suggest that the functional significance may differ between VPS and RAAS in water homeostasis in response to acute stresses affecting body fluids. RAAS would exert more significant functions than VPS in the adaptive response to acute fasting. In this respect, in addition to the reduction in fluid volume, the reduction in sodium intake itself also stimulates RAAS (Boulter et al., 1974; Donckier et al., 1990; Starklint et al., 2005). Accordingly, the restriction of sodium intake as well as of water intake would be involved in triggering RAAS, which further highlights the potential significant contribution of RAAS to water homeostasis under body fluid‐related acute stresses.

Findings of previous studies could provide significant clues on our results that regular exercise and/or rapid restriction did not significantly affect the expression levels of AQP2 protein in the kidney under our experimental conditions. It has been demonstrated that the protein levels of AQP2 were elevated by dehydration (Baggaley et al., 2010; Spector et al., 2002). Conversely, another study reported that the deprivation of food down‐regulated AQP2 protein levels, although the underlying mechanisms still remain unclear (Amlal et al., 2001). In the present study, rats first underwent fasting, followed by additional provision of water. Therefore, it is possible that the first 2 days of fasting would reduce the expression levels of AQP2 protein, and the subsequent dehydration in addition to fasting on the last day could increase the protein levels of AQP2, thereby resulting in comparable levels of AQP2 protein with those at baseline. Future studies are required to address this issue by designing study groups in which fasting or dehydration only with and without regular exercise is conducted.

Significant findings were also obtained for potential factors affecting the expression of SGK‐1. Previous in vivo studies reported that SGK expression was stimulated by aldosterone in both renal medulla and cortex in rats (Bhargava et al., 2001; Shigaev et al., 2000). Another in vitro study demonstrated that the expression of SGK‐1 was also elevated by increased extracellular osmolarity in rat inner medullary collecting duct cells, despite the absence of aldosterone (Chen et al., 2004). Because plasma aldosterone levels and urine osmolarity were elevated by rapid restriction, these factors would contribute to stimulating SGK‐1 expression in the rapid restriction group.

Of note, when rapid restriction was combined with regular exercise, KW and KW/BW ratio were decreased, and plasma creatinine concentration was increased, compared with those of each alone. Although BUN concentration did not differ significantly between the groups, creatinine concentration in the blood has been reported to be a more reliable marker for renal function than BUN, because BUN is more influenced by nonrenal factors (Hosten, 1990). Therefore, these results suggest that rapid restriction with regular exercise has detrimental effects on the kidney, although urine volume and osmolality remained comparable with those observed under rapid restriction alone. We further found that the combination induced renal inflammation, increased the formation of active apoptotic effectors in the kidney, and elevated the levels of early markers for renal injury. In this respect, we argue that aldosterone may partially be implicated in the pathological roles of the combination. It has been reported that, in the principal cells of the cortical collecting duct, aldosterone upregulates SGK‐1 through mineralocorticoid receptor signaling (Leroy et al., 2009). In addition to sodium reabsorption, SGK‐1 stimulates the activation of the proinflammatory transcription factor NF‐κB, thereby leading to the increased expression of proinflammatory cytokines (Leroy et al., 2009). This aldosterone– NF‐κB axis has been assumed to constitute a negative feedback mechanism for attenuating aldosterone‐dependent sodium transport and also to participate in renal inflammation (Leroy et al., 2009). Since circulating levels of aldosterone and expression levels of SGK‐1 were elevated by rapid restriction in our study, it is suggested that rapid restriction stimulates the aldosterone– NF‐κB axis. In addition, the expression profiles of COX‐2 and mPGES‐1, the target genes of NF‐κB (Bogdan et al., 2018; Lee et al., 2010), were similar to those of TNF‐α and IL‐6, which would also support the activation of the aldosterone– NF‐κB axis. Accordingly, aldosterone may have pathological roles in combination‐induced kidney atrophy.

We further found that the expression levels of these NF‐κB target genes and the levels of active apoptotic effectors were particularly elevated in rats subjected to rapid restriction combined with regular exercise. As the levels of aldosterone were comparable between rapid restriction and the combination groups, it is possible that the combination‐derived factors other than aldosterone may exacerbate the renal cytotoxic effects. In this context, our findings suggest the potential increased risk for renal injury by rapid restriction with regular exercise; this could further imply that it would not be desirable for athletes to rapidly reduce their BW by rapid restriction, in terms of kidney integrity. Further investigation is required to elucidate the mechanistic details underlying the potential cytotoxic effects of this combination on the kidney.

This study has some limitations. First, there were no significant differences in BW between the groups with and without rapid restriction for 3 days, although the effects of rapid restriction on urine were evident under our experimental conditions. Accordingly, the present data may represent an earlier phase in the changes or responses affected by rapid restriction, which would correspond to the phase before the manifestation of BW reduction. Additional studies with longer period of restriction would be helpful to gain a comprehensive understanding of the functional significance of VPS and RAAS in water homeostasis. Second, the regular exercise used in this study was the climbing exercise, which is a resistance exercise. It would also be necessary to investigate the effects of aerobic exercises such as treadmill running and swimming on AVP and renal inflammation. Third, the present study did not address the effects of acute recovery from rapid restriction by taking food or water in a short time, as performed by subjects in weight‐sensitive sports. Future studies are needed to tackle these issues using several cohorts with larger sample size, including groups consisting of female rats as well as male rats. It would also be required to examine the effects of longer period of restriction and the impact of the repeated rapid restriction on the future risk for renal dysfunction, including chronic inflammation and fibrosis in the kidney.

In conclusion, to our knowledge, this is the first study to demonstrate the differential functional significance of VPS and RAAS in water homeostasis in response to the rapid restriction of food and water intake with and without regular exercise. Moreover, the combination of rapid restriction and regular exercise‐induced renal inflammation, which would cause renal apoptotic cell death and kidney atrophy. In the natural world, staying in movement irrespective of the lack of food and water would not be rare, and organisms would have to deal with these conditions. Elucidating the mechanisms underlying the response to stresses, including exercise and fasting, would be helpful for developing safe and effective training programs for athletes and also to gain a deeper understanding of the survival strategies of organisms.

## CONFLICT OF INTEREST

The authors declare no conflicts of interest. The results of the present study do not constitute endorsement and are presented clearly, honestly, and without fabrication, falsification, or inappropriate data manipulation.

## AUTHOR CONTRIBUTIONS

KH and MT planned and designed experimental studies. KH and YY performed the experiments and analyzed data. KH and MT drafted the article. All authors read and approved the final manuscript.

## ETHICAL STATEMENT

All the experimental animal handling procedures were approved by the Morioka University on Animal Experimentation.

## SUPPLEMENTARY MATERIALS ARE AVAILABLE AS FOLLOWING LINK

https://figshare.com/s/83e8dfa024d9769ee789
